# *rac*-3-{4-[(2-Hy­droxy­benzyl­idene)amino]-3-phenyl-5-sulfanyl­idene-4,5-dihydro-1*H*-1,2,4-triazol-1-yl}-1,3-diphenyl­propan-1-one

**DOI:** 10.1107/S1600536811035203

**Published:** 2011-09-30

**Authors:** Qing-lei Liu, Wei Wang, Yan Gao, Jing-jing Zhang, Xiao-yu Jia

**Affiliations:** a School of Perfume and Aroma Technology, Shanghai Institute of Technology, Shanghai 200235, People’s Republic of China; b School of Chemical Engineering, University of Science and Technology LiaoNing, Anshan 114051, People’s Republic of China

## Abstract

In the title compound, C_30_H_24_N_4_O_2_S, the four phenyl rings of the substituent groups make dihedral angles of 88.1 (2), 81.0 (2), 21.4 (2) and 44.6 (2)° with the triazole group. An intra­molecular hy­droxy–imino O—H⋯N hydrogen bond results in the formation of an approximately planar (r.m.s deviation = 0.0230 Å) six-membered ring.

## Related literature

For the crystal structures of related 1,2,4-triazole-5(4*H*)-thione derivatives, see: Al-Tamimi *et al.* (2010 ▶); Fun *et al.* (2009 ▶); Gao *et al.* (2011 ▶); Tan *et al.* (2010 ▶); Wang *et al.* (2011 ▶); Zhao *et al.* (2010 ▶).
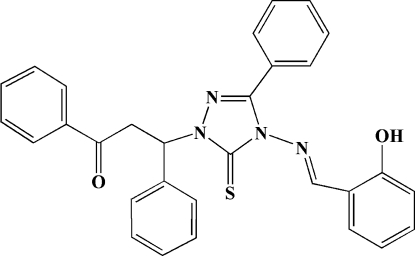


## Experimental

### 

#### Crystal data

C_30_H_24_N_4_O_2_S
*M* *_r_* = 504.59
Triclinic, 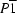

*a* = 6.770 (2) Å
*b* = 13.170 (5) Å
*c* = 14.851 (5) Å
α = 78.114 (12)°
β = 81.715 (15)°
γ = 87.318 (16)°
*V* = 1282.0 (7) Å^3^
*Z* = 2
Mo *K*α radiation
μ = 0.16 mm^−1^
*T* = 113 K
0.20 × 0.18 × 0.12 mm

#### Data collection

Rigaku Saturn CCD area-detector diffractometer
Absorption correction: multi-scan (*CrystalClear*; Rigaku/MSC, 2005 ▶) *T*_min_ = 0.968, *T*_max_ = 0.981
16609 measured reflections
6076 independent reflections
3749 reflections with *I* > 2σ(*I*)
*R*_int_ = 0.043

#### Refinement

*R*[*F* ^2^ > 2σ(*F* ^2^)] = 0.040
*wR*(*F* ^2^) = 0.094
*S* = 0.95
6076 reflections
338 parameters
H atoms treated by a mixture of independent and constrained refinement
Δρ_max_ = 0.29 e Å^−3^
Δρ_min_ = −0.21 e Å^−3^

### 

Data collection: *CrystalClear* (Rigaku/MSC, 2005 ▶); cell refinement: *CrystalClear*; data reduction: *CrystalClear*; program(s) used to solve structure: *SHELXS97* (Sheldrick, 2008 ▶); program(s) used to refine structure: *SHELXL97* (Sheldrick, 2008 ▶); molecular graphics: *SHELXTL* (Sheldrick, 2008 ▶); software used to prepare material for publication: *SHELXTL*.

## Supplementary Material

Crystal structure: contains datablock(s) global, I. DOI: 10.1107/S1600536811035203/zs2139sup1.cif

Structure factors: contains datablock(s) I. DOI: 10.1107/S1600536811035203/zs2139Isup2.hkl

Supplementary material file. DOI: 10.1107/S1600536811035203/zs2139Isup3.cml

Additional supplementary materials:  crystallographic information; 3D view; checkCIF report

## Figures and Tables

**Table 1:** Hydrogen-bond geometry (Å, °)

*D*—H⋯*A*	*D*—H	H⋯*A*	*D*⋯*A*	*D*—H⋯*A*
O2—H2⋯N4	1.00 (3)	1.73 (2)	2.6226 (19)	147.4 (19)

**Fig. 1.**
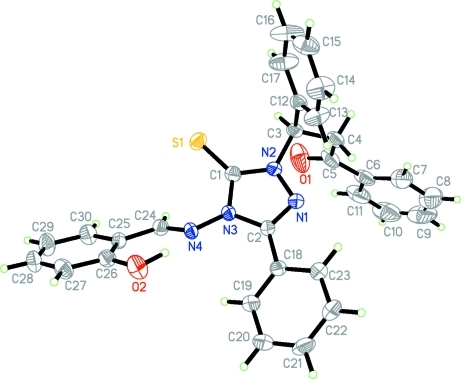
A view of a molecule of the title compound showing the atom-labelling scheme. Displacement ellipsoids are drawn at the 55% probability level.

## supplementary crystallographic information

### Comment

In a continuation of the structural study by our group (Wang *et al.*, 2011) of Mannich base derivatives synthesized by reactions of amino heterocycles and aromatic aldehydes, we present here the crystal structure of the title compound, the substituted 1,2,4-triazole-5(4*H*)-thione derivative C30 H24 N4 O2 S. The bond lengths and angles in this compound are found to have normal values, comparable with those reported in the related 1,2,4-triazole- 5(4*H*)-thione derivatives (Al-Tamimi *et al.*, 2010; Fun *et al.* (2009); Tan *et al.* (2010); Wang *et al.*, 2011;). The C1 and C2 atoms in the triazole ring show distorted C*_sp_*^2^ hybridization states with the bond angles of 102.24 (11)° (N2—C1—N3); 130.08 (11)° (N3—C1—S1); 109.77 (12)° (N1—C2—N3) and 127.77 (12) ° (N3—C2—C18), which are similar to those in the reported triazole derivatives (Zhao *et al.*, 2010; Gao *et al.*, 2011). There are four phenyl rings in the molecular structure. The four phenyl rings of the substituent groups (C1–C6, C12–C17, C18–C23 and C25–C30) make dihedral angles of 88.1 (2), 81.0 (2), 21.4 (2) and 135.4 (2)°, respectively, with the triazole group.

An intramolecular hydroxyl O—H···N hydrogen bond with the imino group results in the formation of a planar six-membered ring [r.m.s deviation = 0.0230 (2) Å].

### Experimental

The title compound was synthesized with the reaction of 2-hydroxybenzaldehyde (2.0 mmol) and 3-(4-amino-3-phenyl-5-thioxo-4,5- dihydro-1*H*-1,2,4-triazol-1-yl)-1,3-diphenylpropan-1-one (2.0 mmol) by refluxing in ethanol. The reaction progress was monitored *via* TLC. The resulting precipitate was filtered off, washed with cold ethanol, dried and purified to give the target product as a colorless solid in 80% yield. Crystals suitable for single-crystal X-ray analysis were grown by slow evaporation of a solution in chloroform–ethanol (1:1).

### Refinement

The H atom of the hydroxy group was located in a difference electron density map and the atomic coordinates and isotropic thermal displacement parameter were allowed to refine freely. Other H atoms were positioned geometrically and refined as riding (C—H = 0.95–1.00 Å) on their parent atoms, with *U*_iso_(H) = 1.2*U*_eq_(C).

### Crystal data

**Table:**

C_30_H_24_N_4_O_2_S	*Z* = 2
*M**_r_* = 504.59	*F*(000) = 528
Triclinic, *P*1	*D*_x_ = 1.307 Mg m^−^^3^
Hall symbol: -P 1	Mo *K*α radiation, λ = 0.71073 Å
*a* = 6.770 (2) Å	Cell parameters from 4176 reflections
*b* = 13.170 (5) Å	θ = 1.4–28.1°
*c* = 14.851 (5) Å	µ = 0.16 mm^−^^1^
α = 78.114 (12)°	*T* = 113 K
β = 81.715 (15)°	Prism, colorless
γ = 87.318 (16)°	0.20 × 0.18 × 0.12 mm
*V* = 1282.0 (7) Å^3^	

### Data collection

**Table:**

Rigaku Saturn CCD area-detector diffractometer	6076 independent reflections
Radiation source: rotating anode	3749 reflections with *I* > 2σ(*I*)
multilayer	*R*_int_ = 0.043
Detector resolution: 14.63 pixels mm^-1^	θ_max_ = 27.9°, θ_min_ = 1.4°
φ and ω scans	*h* = −8→8
Absorption correction: multi-scan (*CrystalClear*; Rigaku/MSC, 2005)	*k* = −17→17
*T*_min_ = 0.968, *T*_max_ = 0.981	*l* = −18→19
16609 measured reflections	

### Refinement

**Table:**

Refinement on *F*^2^	Primary atom site location: structure-invariant direct methods
Least-squares matrix: full	Secondary atom site location: difference Fourier map
*R*[*F*^2^ > 2σ(*F*^2^)] = 0.040	Hydrogen site location: inferred from neighbouring sites
*wR*(*F*^2^) = 0.094	H atoms treated by a mixture of independent and constrained refinement
*S* = 0.95	*w* = 1/[σ^2^(*F*_o_^2^) + (0.0415*P*)^2^] where *P* = (*F*_o_^2^ + 2*F*_c_^2^)/3
6076 reflections	(Δ/σ)_max_ = 0.001
338 parameters	Δρ_max_ = 0.29 e Å^−^^3^
0 restraints	Δρ_min_ = −0.21 e Å^−^^3^

### Special details

**Table:**

Geometry. All e.s.d.'s (except the e.s.d. in the dihedral angle between two l.s. planes) are estimated using the full covariance matrix. The cell e.s.d.'s are taken into account individually in the estimation of e.s.d.'s in distances, angles and torsion angles; correlations between e.s.d.'s in cell parameters are only used when they are defined by crystal symmetry. An approximate (isotropic) treatment of cell e.s.d.'s is used for estimating e.s.d.'s involving l.s. planes.
Refinement. Refinement of *F*^2^ against ALL reflections. The weighted *R*-factor *wR* and goodness of fit *S* are based on *F*^2^, conventional *R*-factors *R* are based on *F*, with *F* set to zero for negative *F*^2^. The threshold expression of *F*^2^ > σ(*F*^2^) is used only for calculating *R*-factors(gt) *etc*. and is not relevant to the choice of reflections for refinement. *R*-factors based on *F*^2^ are statistically about twice as large as those based on *F*, and *R*- factors based on ALL data will be even larger.

### Fractional atomic coordinates and isotropic or equivalent isotropic displacement parameters (Å^2^)

**Table:**

	*x*	*y*	*z*	*U*_iso_*/*U*_eq_	
S1	1.23731 (6)	0.74106 (3)	0.66002 (2)	0.02837 (11)	
O1	1.1833 (2)	0.82091 (11)	0.92569 (8)	0.0578 (4)	
O2	0.83353 (15)	0.41453 (9)	0.60325 (8)	0.0347 (3)	
H2	0.828 (3)	0.470 (2)	0.6402 (16)	0.113 (9)*	
N1	0.75550 (17)	0.71629 (9)	0.84047 (8)	0.0248 (3)	
N2	0.92480 (17)	0.76425 (9)	0.79086 (7)	0.0227 (3)	
N3	0.92676 (16)	0.61510 (9)	0.75493 (7)	0.0212 (3)	
N4	0.95198 (17)	0.53608 (9)	0.70367 (7)	0.0224 (3)	
C1	1.0334 (2)	0.70663 (11)	0.73499 (9)	0.0223 (3)	
C2	0.7565 (2)	0.62593 (11)	0.81636 (9)	0.0218 (3)	
C3	0.9499 (2)	0.87572 (11)	0.78459 (9)	0.0233 (3)	
H3	1.0950	0.8915	0.7666	0.028*	
C4	0.8830 (2)	0.90266 (11)	0.87934 (9)	0.0282 (3)	
H4A	0.8801	0.9790	0.8728	0.034*	
H4B	0.7457	0.8776	0.9023	0.034*	
C5	1.0196 (3)	0.85503 (12)	0.95006 (11)	0.0355 (4)	
C6	0.9467 (3)	0.85086 (12)	1.05085 (10)	0.0382 (4)	
C7	0.7628 (3)	0.89039 (14)	1.08254 (11)	0.0455 (5)	
H7	0.6782	0.9243	1.0394	0.055*	
C8	0.7012 (3)	0.88071 (16)	1.17744 (12)	0.0597 (6)	
H8	0.5743	0.9072	1.1993	0.072*	
C9	0.8262 (4)	0.83231 (17)	1.23954 (13)	0.0711 (7)	
H9	0.7835	0.8245	1.3043	0.085*	
C10	1.0123 (4)	0.79515 (16)	1.20885 (13)	0.0680 (7)	
H10	1.0989	0.7638	1.2521	0.082*	
C11	1.0712 (3)	0.80400 (13)	1.11513 (11)	0.0518 (5)	
H11	1.1986	0.7778	1.0938	0.062*	
C12	0.8368 (2)	0.93823 (11)	0.71014 (9)	0.0240 (3)	
C13	0.6371 (2)	0.92297 (14)	0.70853 (11)	0.0398 (4)	
H13	0.5671	0.8718	0.7553	0.048*	
C14	0.5375 (3)	0.98140 (14)	0.63950 (12)	0.0455 (5)	
H14	0.4007	0.9690	0.6389	0.055*	
C15	0.6345 (3)	1.05683 (13)	0.57213 (11)	0.0400 (4)	
H15	0.5645	1.0986	0.5264	0.048*	
C16	0.8329 (3)	1.07093 (14)	0.57177 (12)	0.0521 (5)	
H16	0.9025	1.1215	0.5243	0.062*	
C17	0.9338 (3)	1.01215 (14)	0.64007 (11)	0.0447 (5)	
H17	1.0721	1.0229	0.6386	0.054*	
C18	0.5920 (2)	0.55264 (11)	0.84967 (9)	0.0232 (3)	
C19	0.6105 (2)	0.44711 (12)	0.84903 (10)	0.0325 (4)	
H19	0.7353	0.4187	0.8268	0.039*	
C20	0.4476 (2)	0.38335 (13)	0.88066 (11)	0.0381 (4)	
H20	0.4609	0.3115	0.8794	0.046*	
C21	0.2664 (2)	0.42313 (13)	0.91398 (10)	0.0350 (4)	
H21	0.1550	0.3791	0.9356	0.042*	
C22	0.2481 (2)	0.52800 (13)	0.91564 (10)	0.0318 (4)	
H22	0.1237	0.5556	0.9392	0.038*	
C23	0.4081 (2)	0.59262 (12)	0.88353 (9)	0.0267 (3)	
H23	0.3933	0.6645	0.8844	0.032*	
C24	1.1334 (2)	0.50562 (11)	0.68280 (9)	0.0227 (3)	
H24	1.2383	0.5311	0.7077	0.027*	
C25	1.1778 (2)	0.43255 (11)	0.62150 (9)	0.0221 (3)	
C26	1.0299 (2)	0.39283 (11)	0.58138 (10)	0.0241 (3)	
C27	1.0835 (2)	0.32828 (12)	0.51852 (10)	0.0300 (4)	
H27	0.9836	0.3018	0.4912	0.036*	
C28	1.2809 (2)	0.30257 (12)	0.49579 (10)	0.0338 (4)	
H28	1.3162	0.2587	0.4524	0.041*	
C29	1.4298 (2)	0.33974 (13)	0.53524 (11)	0.0358 (4)	
H29	1.5658	0.3209	0.5198	0.043*	
C30	1.3763 (2)	0.40458 (12)	0.59726 (10)	0.0303 (4)	
H30	1.4773	0.4308	0.6240	0.036*	

### Atomic displacement parameters (Å^2^)

**Table:**

	*U*^11^	*U*^22^	*U*^33^	*U*^12^	*U*^13^	*U*^23^
S1	0.0274 (2)	0.0312 (2)	0.0263 (2)	−0.00792 (17)	0.00275 (16)	−0.00803 (16)
O1	0.0705 (9)	0.0658 (10)	0.0453 (8)	0.0350 (8)	−0.0258 (7)	−0.0258 (7)
O2	0.0215 (6)	0.0371 (7)	0.0515 (7)	0.0004 (5)	−0.0089 (5)	−0.0206 (6)
N1	0.0280 (7)	0.0198 (6)	0.0251 (6)	−0.0032 (5)	0.0028 (5)	−0.0047 (5)
N2	0.0278 (7)	0.0175 (6)	0.0221 (6)	−0.0035 (5)	0.0009 (5)	−0.0047 (5)
N3	0.0226 (7)	0.0191 (6)	0.0225 (6)	−0.0021 (5)	0.0007 (5)	−0.0078 (5)
N4	0.0251 (7)	0.0198 (6)	0.0236 (6)	−0.0014 (5)	0.0000 (5)	−0.0096 (5)
C1	0.0259 (8)	0.0215 (8)	0.0205 (7)	−0.0013 (6)	−0.0057 (6)	−0.0049 (6)
C2	0.0240 (8)	0.0194 (7)	0.0207 (7)	0.0012 (6)	0.0005 (6)	−0.0040 (6)
C3	0.0277 (8)	0.0173 (7)	0.0253 (8)	−0.0048 (6)	−0.0026 (6)	−0.0047 (6)
C4	0.0402 (9)	0.0197 (8)	0.0260 (8)	0.0002 (7)	−0.0060 (7)	−0.0072 (6)
C5	0.0568 (12)	0.0214 (8)	0.0319 (9)	0.0065 (8)	−0.0132 (8)	−0.0101 (7)
C6	0.0720 (13)	0.0184 (8)	0.0267 (9)	−0.0022 (8)	−0.0124 (9)	−0.0060 (7)
C7	0.0737 (14)	0.0363 (10)	0.0284 (9)	−0.0090 (10)	−0.0047 (9)	−0.0107 (8)
C8	0.0915 (17)	0.0539 (13)	0.0347 (11)	−0.0183 (12)	0.0057 (11)	−0.0172 (10)
C9	0.137 (2)	0.0507 (14)	0.0249 (10)	−0.0321 (15)	−0.0065 (13)	−0.0031 (10)
C10	0.132 (2)	0.0381 (12)	0.0351 (11)	−0.0064 (14)	−0.0262 (13)	0.0014 (9)
C11	0.0972 (16)	0.0258 (10)	0.0354 (10)	0.0024 (10)	−0.0230 (10)	−0.0042 (8)
C12	0.0305 (9)	0.0191 (7)	0.0238 (7)	−0.0030 (6)	−0.0042 (6)	−0.0067 (6)
C13	0.0358 (10)	0.0415 (11)	0.0363 (9)	−0.0082 (8)	−0.0037 (8)	0.0066 (8)
C14	0.0349 (10)	0.0502 (12)	0.0492 (11)	−0.0031 (9)	−0.0146 (8)	0.0018 (9)
C15	0.0531 (12)	0.0288 (9)	0.0403 (10)	0.0004 (8)	−0.0211 (9)	−0.0020 (8)
C16	0.0608 (13)	0.0387 (11)	0.0505 (11)	−0.0201 (10)	−0.0215 (10)	0.0191 (9)
C17	0.0413 (11)	0.0404 (11)	0.0488 (11)	−0.0176 (9)	−0.0163 (9)	0.0102 (9)
C18	0.0278 (8)	0.0205 (8)	0.0205 (7)	−0.0030 (6)	−0.0005 (6)	−0.0036 (6)
C19	0.0320 (9)	0.0239 (8)	0.0378 (9)	−0.0032 (7)	0.0070 (7)	−0.0051 (7)
C20	0.0455 (11)	0.0235 (9)	0.0433 (10)	−0.0108 (8)	0.0063 (8)	−0.0082 (7)
C21	0.0347 (10)	0.0381 (10)	0.0300 (9)	−0.0178 (8)	0.0036 (7)	−0.0037 (7)
C22	0.0268 (9)	0.0382 (10)	0.0283 (8)	−0.0036 (7)	0.0030 (7)	−0.0058 (7)
C23	0.0283 (9)	0.0254 (8)	0.0252 (8)	−0.0012 (7)	0.0002 (6)	−0.0048 (6)
C24	0.0224 (8)	0.0233 (8)	0.0232 (7)	−0.0013 (6)	−0.0033 (6)	−0.0061 (6)
C25	0.0220 (8)	0.0226 (8)	0.0220 (7)	−0.0013 (6)	−0.0005 (6)	−0.0067 (6)
C26	0.0222 (8)	0.0237 (8)	0.0267 (8)	−0.0010 (6)	−0.0033 (6)	−0.0057 (6)
C27	0.0351 (9)	0.0273 (9)	0.0314 (8)	−0.0008 (7)	−0.0103 (7)	−0.0110 (7)
C28	0.0414 (10)	0.0326 (9)	0.0296 (8)	0.0026 (8)	−0.0004 (7)	−0.0150 (7)
C29	0.0253 (9)	0.0417 (10)	0.0428 (10)	0.0041 (8)	0.0012 (7)	−0.0193 (8)
C30	0.0223 (8)	0.0345 (9)	0.0370 (9)	−0.0011 (7)	−0.0041 (7)	−0.0136 (7)

### Geometric parameters (Å, °)

**Table:**

S1—C1	1.6608 (15)	C13—C14	1.387 (2)
O1—C5	1.2104 (19)	C13—H13	0.9500
O2—C26	1.3538 (17)	C14—C15	1.371 (2)
O2—H2	1.00 (2)	C14—H14	0.9500
N1—C2	1.3110 (17)	C15—C16	1.364 (2)
N1—N2	1.3741 (16)	C15—H15	0.9500
N2—C1	1.3551 (17)	C16—C17	1.386 (2)
N2—C3	1.4681 (18)	C16—H16	0.9500
N3—C2	1.3841 (17)	C17—H17	0.9500
N3—C1	1.3910 (18)	C18—C19	1.392 (2)
N3—N4	1.4015 (15)	C18—C23	1.3958 (19)
N4—C24	1.2890 (17)	C19—C20	1.385 (2)
C2—C18	1.4703 (19)	C19—H19	0.9500
C3—C12	1.516 (2)	C20—C21	1.377 (2)
C3—C4	1.5225 (19)	C20—H20	0.9500
C3—H3	1.0000	C21—C22	1.386 (2)
C4—C5	1.519 (2)	C21—H21	0.9500
C4—H4A	0.9900	C22—C23	1.377 (2)
C4—H4B	0.9900	C22—H22	0.9500
C5—C6	1.498 (2)	C23—H23	0.9500
C6—C7	1.383 (2)	C24—C25	1.4493 (19)
C6—C11	1.394 (2)	C24—H24	0.9500
C7—C8	1.392 (2)	C25—C30	1.3923 (19)
C7—H7	0.9500	C25—C26	1.4076 (19)
C8—C9	1.380 (3)	C26—C27	1.3878 (19)
C8—H8	0.9500	C27—C28	1.375 (2)
C9—C10	1.380 (3)	C27—H27	0.9500
C9—H9	0.9500	C28—C29	1.391 (2)
C10—C11	1.374 (2)	C28—H28	0.9500
C10—H10	0.9500	C29—C30	1.381 (2)
C11—H11	0.9500	C29—H29	0.9500
C12—C13	1.380 (2)	C30—H30	0.9500
C12—C17	1.381 (2)		
			
C26—O2—H2	105.6 (13)	C14—C13—H13	119.6
C2—N1—N2	105.10 (11)	C15—C14—C13	120.74 (16)
C1—N2—N1	113.68 (11)	C15—C14—H14	119.6
C1—N2—C3	124.75 (12)	C13—C14—H14	119.6
N1—N2—C3	119.92 (11)	C16—C15—C14	118.96 (16)
C2—N3—C1	109.06 (11)	C16—C15—H15	120.5
C2—N3—N4	122.82 (11)	C14—C15—H15	120.5
C1—N3—N4	125.91 (11)	C15—C16—C17	120.56 (16)
C24—N4—N3	115.83 (12)	C15—C16—H16	119.7
N2—C1—N3	102.24 (11)	C17—C16—H16	119.7
N2—C1—S1	127.65 (11)	C12—C17—C16	121.15 (16)
N3—C1—S1	130.08 (11)	C12—C17—H17	119.4
N1—C2—N3	109.77 (12)	C16—C17—H17	119.4
N1—C2—C18	122.42 (12)	C19—C18—C23	119.04 (14)
N3—C2—C18	127.77 (12)	C19—C18—C2	123.69 (14)
N2—C3—C12	110.20 (11)	C23—C18—C2	117.27 (13)
N2—C3—C4	109.44 (11)	C20—C19—C18	120.17 (15)
C12—C3—C4	112.21 (12)	C20—C19—H19	119.9
N2—C3—H3	108.3	C18—C19—H19	119.9
C12—C3—H3	108.3	C21—C20—C19	120.59 (16)
C4—C3—H3	108.3	C21—C20—H20	119.7
C5—C4—C3	112.33 (13)	C19—C20—H20	119.7
C5—C4—H4A	109.1	C20—C21—C22	119.37 (15)
C3—C4—H4A	109.1	C20—C21—H21	120.3
C5—C4—H4B	109.1	C22—C21—H21	120.3
C3—C4—H4B	109.1	C23—C22—C21	120.75 (15)
H4A—C4—H4B	107.9	C23—C22—H22	119.6
O1—C5—C6	120.73 (15)	C21—C22—H22	119.6
O1—C5—C4	120.89 (14)	C22—C23—C18	120.07 (15)
C6—C5—C4	118.38 (15)	C22—C23—H23	120.0
C7—C6—C11	119.05 (16)	C18—C23—H23	120.0
C7—C6—C5	123.21 (15)	N4—C24—C25	120.16 (13)
C11—C6—C5	117.73 (17)	N4—C24—H24	119.9
C6—C7—C8	120.28 (18)	C25—C24—H24	119.9
C6—C7—H7	119.9	C30—C25—C26	118.44 (13)
C8—C7—H7	119.9	C30—C25—C24	118.69 (13)
C9—C8—C7	119.4 (2)	C26—C25—C24	122.78 (13)
C9—C8—H8	120.3	O2—C26—C27	118.16 (13)
C7—C8—H8	120.3	O2—C26—C25	121.82 (13)
C10—C9—C8	120.97 (19)	C27—C26—C25	120.02 (13)
C10—C9—H9	119.5	C28—C27—C26	120.05 (14)
C8—C9—H9	119.5	C28—C27—H27	120.0
C11—C10—C9	119.3 (2)	C26—C27—H27	120.0
C11—C10—H10	120.4	C27—C28—C29	121.10 (15)
C9—C10—H10	120.4	C27—C28—H28	119.4
C10—C11—C6	121.0 (2)	C29—C28—H28	119.4
C10—C11—H11	119.5	C30—C29—C28	118.73 (15)
C6—C11—H11	119.5	C30—C29—H29	120.6
C13—C12—C17	117.74 (14)	C28—C29—H29	120.6
C13—C12—C3	122.08 (13)	C29—C30—C25	121.65 (14)
C17—C12—C3	120.17 (14)	C29—C30—H30	119.2
C12—C13—C14	120.78 (15)	C25—C30—H30	119.2
C12—C13—H13	119.6		
			
C2—N1—N2—C1	0.44 (15)	N2—C3—C12—C13	52.78 (19)
C2—N1—N2—C3	166.47 (11)	C4—C3—C12—C13	−69.45 (18)
C2—N3—N4—C24	−150.62 (12)	N2—C3—C12—C17	−126.17 (15)
C1—N3—N4—C24	48.15 (18)	C4—C3—C12—C17	111.60 (16)
N1—N2—C1—N3	−2.54 (15)	C17—C12—C13—C14	−1.0 (2)
C3—N2—C1—N3	−167.79 (12)	C3—C12—C13—C14	179.99 (15)
N1—N2—C1—S1	175.64 (10)	C12—C13—C14—C15	−1.1 (3)
C3—N2—C1—S1	10.4 (2)	C13—C14—C15—C16	2.6 (3)
C2—N3—C1—N2	3.62 (14)	C14—C15—C16—C17	−2.0 (3)
N4—N3—C1—N2	167.00 (12)	C13—C12—C17—C16	1.6 (3)
C2—N3—C1—S1	−174.50 (11)	C3—C12—C17—C16	−179.37 (16)
N4—N3—C1—S1	−11.1 (2)	C15—C16—C17—C12	−0.1 (3)
N2—N1—C2—N3	1.96 (15)	N1—C2—C18—C19	−160.38 (14)
N2—N1—C2—C18	−175.88 (12)	N3—C2—C18—C19	22.2 (2)
C1—N3—C2—N1	−3.66 (16)	N1—C2—C18—C23	19.7 (2)
N4—N3—C2—N1	−167.66 (12)	N3—C2—C18—C23	−157.76 (13)
C1—N3—C2—C18	174.03 (13)	C23—C18—C19—C20	0.7 (2)
N4—N3—C2—C18	10.0 (2)	C2—C18—C19—C20	−179.24 (13)
C1—N2—C3—C12	82.90 (17)	C18—C19—C20—C21	−0.7 (2)
N1—N2—C3—C12	−81.50 (15)	C19—C20—C21—C22	0.0 (2)
C1—N2—C3—C4	−153.26 (13)	C20—C21—C22—C23	0.7 (2)
N1—N2—C3—C4	42.35 (16)	C21—C22—C23—C18	−0.7 (2)
N2—C3—C4—C5	67.58 (16)	C19—C18—C23—C22	0.0 (2)
C12—C3—C4—C5	−169.76 (12)	C2—C18—C23—C22	179.93 (13)
C3—C4—C5—O1	15.4 (2)	N3—N4—C24—C25	−173.25 (12)
C3—C4—C5—C6	−164.39 (14)	N4—C24—C25—C30	177.34 (13)
O1—C5—C6—C7	178.74 (16)	N4—C24—C25—C26	0.9 (2)
C4—C5—C6—C7	−1.5 (2)	C30—C25—C26—O2	178.39 (13)
O1—C5—C6—C11	−1.7 (2)	C24—C25—C26—O2	−5.1 (2)
C4—C5—C6—C11	178.03 (14)	C30—C25—C26—C27	−0.7 (2)
C11—C6—C7—C8	−1.8 (3)	C24—C25—C26—C27	175.84 (13)
C5—C6—C7—C8	177.74 (16)	O2—C26—C27—C28	−178.70 (13)
C6—C7—C8—C9	0.7 (3)	C25—C26—C27—C28	0.4 (2)
C7—C8—C9—C10	1.2 (3)	C26—C27—C28—C29	0.4 (2)
C8—C9—C10—C11	−1.9 (3)	C27—C28—C29—C30	−0.9 (2)
C9—C10—C11—C6	0.7 (3)	C28—C29—C30—C25	0.6 (2)
C7—C6—C11—C10	1.1 (3)	C26—C25—C30—C29	0.2 (2)
C5—C6—C11—C10	−178.46 (17)	C24—C25—C30—C29	−176.46 (14)

### Hydrogen-bond geometry (Å, °)

**Table:**

*D*—H···*A*	*D*—H	H···*A*	*D*···*A*	*D*—H···*A*
O2—H2···N4	1.00 (3)	1.73 (2)	2.6226 (19)	147.4 (19)
